# Interferon-Induced Protein 6 (IFI6): Biological Functions, Molecular Mechanisms and Pathological Roles in Human Diseases

**DOI:** 10.3390/ijms27209340

**Published:** 2026-10-21

**Authors:** Jingyu Yang, Yuehong Chen, Yulin Zhang, Qibing Xie, Geng Yin

**Affiliations:** 1 West China School of Medicine, Sichuan University, Chengdu 610041, China; 2 Department of Rheumatology and Immunology, West China Hospital, Sichuan University, 37 Guoxue Lane, Chengdu 610041, China

**Keywords:** IFI6, immune regulation, tumor progression, viral infection

## Abstract

Interferon (IFN)-induced protein 6 (IFI6) is an important member of the IFN-stimulated gene (ISG) family. It plays significant roles in anti-apoptosis, immune regulation, maintenance of mitochondrial homeostasis, antiviral defense, and tumor progression. At the molecular mechanism level, IFI6 inhibits mitochondrial membrane depolarization, cytochrome c release, and caspase activation. It also regulates the balance between B-cell lymphoma 2 (Bcl-2)/Bcl-2 interacting mediator of cell death (Bim), activates the Janus kinase (JAK)/signal transducer and activator of transcription (STAT) and phosphatidylinositol 3-kinase-protein kinase B (PI3K-AKT) pathways, and upregulates the ferroptosis-related proteins solute carrier family 7 member 11 (SLC7A11) and glutathione peroxidase 4 (GPX4), thereby enhancing cell survival and proliferation through multiple pathways. In terms of immune regulation, IFI6 is associated with immune cells (such as T/B cells, etc.) and the tumor microenvironment. IFI6 regulates mitochondrial reactive oxygen species (mtROS) and mitochondrial calcium homeostasis, influencing respiratory chain assembly and metabolism. In terms of viral infection, IFI6 may inhibit the replication of multiple viruses (such as hepatitis B virus (HBV), hepatitis C virus (HCV), SARS-CoV-2, etc.) by inhibiting flavivirus-induced endoplasmic reticulum (ER) invagination, interfering with retinoic acid-inducible gene I (RIG-I)-like receptor signaling pathways, and inhibiting epidermal growth factor receptor (EGFR) kinase activity. In addition, IFI6 is highly expressed in autoimmune diseases and malignant tumors and is associated with proliferation, metastasis, and poor prognosis. In this review, we aim to highlight IFI6 as a potential key target in disease diagnosis and treatment.

## 1. Introduction

The interferon (IFN) system is a core component of the body’s innate immune defense and plays an irreplaceable role in resisting viral infections and regulating anti-tumor immunity. When pathogens invade, pattern recognition receptors (PRRs) on the surface of host cells recognize pathogen-associated molecular patterns (PAMPs), activating downstream signaling cascades and inducing the production and secretion of type I IFNs. After binding to their cell surface receptors, type I IFNs initiate the transcription of IFN-stimulated genes (ISGs). The protein products encoded by these ISGs directly or indirectly perform various biological functions such as antiviral defense, immune regulation, and the regulation of cell survival, death and proliferation [1]. As one of the ISGs, IFN-induced protein 6 (IFI6) has achieved significant progress in functional research, and the complexity of its biological functions is gradually being revealed. However, its precise mechanisms of action under different pathological conditions, its interactive regulatory networks with other signaling pathways, and the feasibility and safety of targeting it as a therapeutic target still require in-depth investigation. Although relevant basic research exists, the clinical value of IFI6 in autoimmune diseases, tumors, and viral infections remains to be validated. This study systematically reviews the structure, biological functions, and research progress of IFI6 in various human diseases, providing a theoretical reference for subsequent basic mechanistic research and clinical translation.

## 2. Structure of IFI6

IFI6, also known as IFI6-16 and G1P3 (ISG 6-16), belongs to the FAM14 family and is one of the ISGs. Located on the short arm of chromosome 1 at 1p35, IFI6 encodes a 130-amino-acid mitochondria-related protein that is induced and upregulated by type I IFNs, including IFN-α and IFN-β. It plays important roles in various biological functions such as regulating apoptosis and immunity. In recent years, multiple studies have gradually revealed its important roles in various diseases, such as autoimmune diseases, tumors, and viral infectious diseases [2].

The protein structure of IFI6 consists sequentially of a 21-amino-acid N-terminal signal peptide, followed by a 19-amino-acid hydrophilic region 1 (HL-1), a 20-amino-acid transmembrane region 1 (TM-1), a linker region (L), a 20-amino-acid transmembrane region 2 (TM-2), and a 34-amino-acid hydrophilic region 2 (HL-2). This dual transmembrane-dual hydrophilic structural pattern is highly conserved in the FAM14 family [3]. Surface probability analysis by Emini’s method, which indicates the probability of a region being located on the protein surface, revealed that the signal peptide and HL-1 and HL-2 regions are more easily exposed to solvent, suggesting that these regions may be key sites for protein–protein interactions. The polar HL-1 may form an extended random coil, while HL-2 may form an amphipathic helix. Since two transmembrane domains separate HL-1 and HL-2, they may not form well-folded domains but instead protrude as unfolded rope-like structures for binding by other proteins [3].

Early studies found that IFI6 is mainly localized to the inner mitochondrial membrane and co-localizes with cytochrome c. Recent studies have shown that IFI6 is an endoplasmic reticulum (ER)-integrated membrane protein. IFI6 is highly hydrophobic and may possess transmembrane helices, suggesting a potential association with the membrane, although it cannot be ruled out that some proteins may bind to the membrane through peripheral interactions [4]. This seemingly contradictory localization result may reflect the dynamic subcellular distribution of IFI6 across different cell types, stimulation conditions, or time points, and this dynamic subcellular localization may be closely related to its multiple biological functions.

## 3. Biological Functions of IFI6

**Figure 1:**
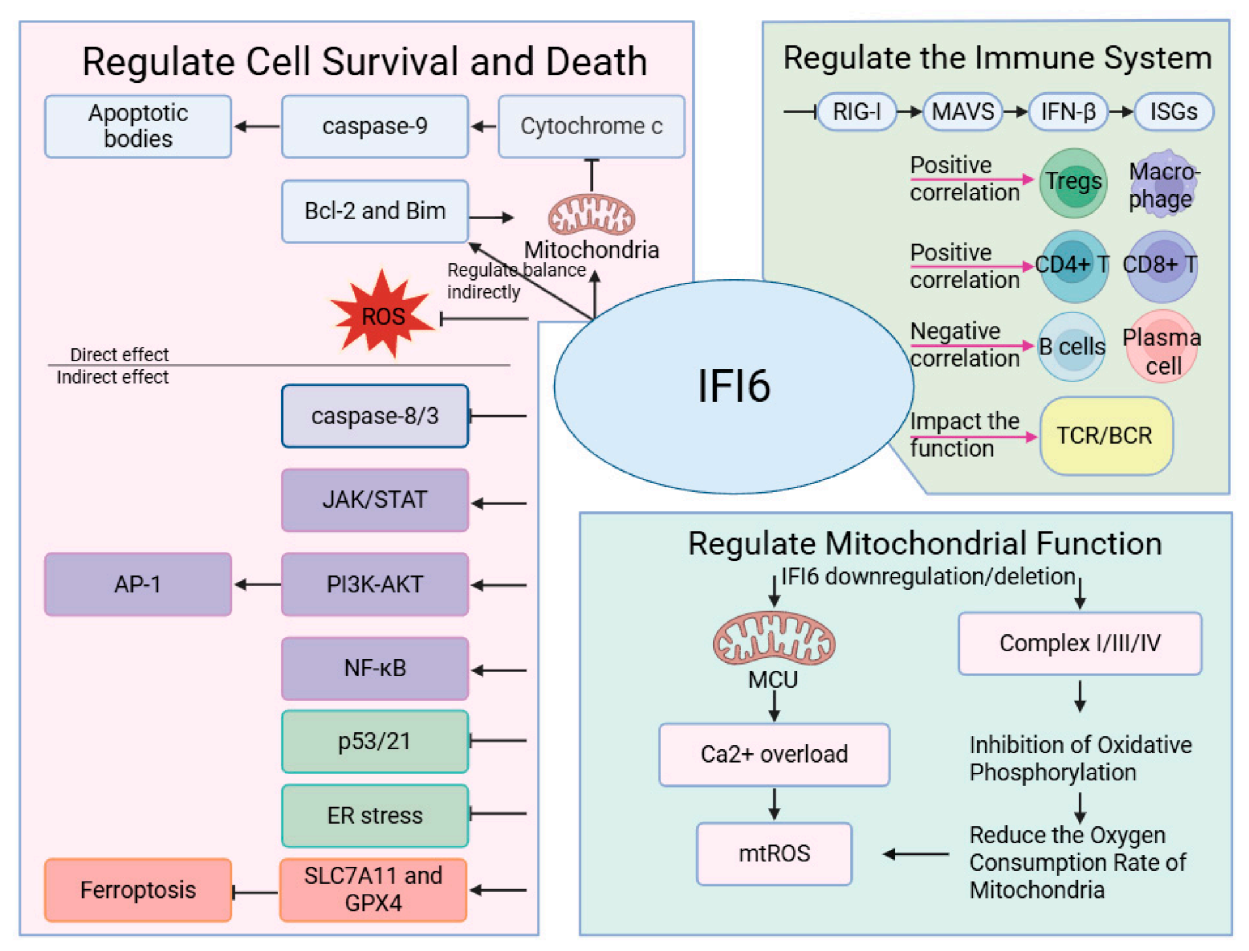
The biological function of IFI6. Bcl-2: B-cell lymphoma 2, Bim: Bcl-2 interacting mediator of cell death, ROS: reactive oxygen species, JAK: Janus kinase, STAT: signal transducer and activator of transcription, AP-1: activator protein 1, PI3K: phosphoinositide 3-kinase, AKT: protein kinase B, NF-κB: nuclear factor kappa B, ER: endoplasmic reticulum, SLC7A11: solute carrier family 7 member 11, GPX4: glutathione peroxidase 4, RIG-I: retinoic acid-inducible gene I, MAVS: mitochondrial antiviral signaling protein, IFN: interferon, ISGs: interferon-stimulated genes, TCR: T cell receptor, BCR: B cell receptor, MCU: mitochondrial Ca^2+^ uniporter, mtROS: mitochondrial reactive oxygen species.

Although the biological functions of IFI6 remain incompletely understood, IFI6, as a multifunctional IFN-stimulated gene, primarily exerts its biological functions through three interrelated dimensions: regulation of cell survival and death, immune regulation, and mitochondrial function regulation (Figure 1). In terms of anti-apoptosis, IFI6 promotes cell survival through multiple mechanisms including inhibiting mitochondrial membrane depolarization to block cytochrome c release and caspase activation, activating the Janus kinase (JAK)/signal transducer and activator of transcription (STAT) and phosphatidylinositol 3-kinase–protein kinase B (PI3K-AKT) pathways, and inhibiting ferroptosis. In terms of immune regulation, IFI6 acts as a negative-feedback factor of the retinoic acid-inducible gene I (RIG-I) signaling pathway to inhibit excessive IFN responses, and is associated with the functional regulation of Tregs, CD4^+^ T cells, and B cells. In terms of mitochondrial function, IFI6 affects respiratory chain complex assembly and cell metabolism by regulating mitochondrial reactive oxygen species (mtROS) production and mitochondrial calcium homeostasis. Through these functions, IFI6 plays important pathological regulatory roles in tumors, autoimmune diseases, and viral infections [5,6].

### 3.1. Regulate Cell Survival and Death

#### 3.1.1. Anti-Apoptosis

Inhibition of the mitochondrial apoptotic pathway. IFI6 is mainly enriched in the inner mitochondrial membrane and co-localizes with cytochrome c. Under apoptotic signal stimulation, mitochondrial outer membrane permeability increases; cytochrome c is released into the cytoplasm, binds with apoptotic protease activating factor-1 (Apaf-1), causing it to form polymers, and promotes caspase-9 binding to form apoptosomes, thereby activating caspase-3 and inducing apoptosis. IFI6 maintains the integrity of the mitochondrial outer membrane by inhibiting mitochondrial membrane depolarization, thereby blocking cytochrome c release, inhibiting caspase-3 activity, or delaying apoptotic signals initiated by the tumor necrosis factor (TNF)-related apoptosis-inducing ligand (TRAIL)/caspase-8 pathway, thus inhibiting apoptosis [7]. Studies have found that IFI6 can regulate the balance between Bcl-2 and Bim expression, stabilize mitochondria, and thereby resist apoptosis. Meanwhile, studies have revealed that mitochondrial Ca^2+^ overload may induce tumor cell apoptosis through IFI6 downregulation [8]. IFI6 knockdown increases reactive oxygen species (ROS) accumulation, thereby inhibiting cancer cell proliferation and inducing apoptosis; similarly, high IFI6 expression can inhibit apoptosis [2]. In esophageal squamous cell carcinoma (ESCC), IFI6 silencing significantly promoted mtROS generation, and the ROS inhibitor N-acetylcysteine reversed IFI6 silencing-induced apoptosis, strongly demonstrating the role of IFI6 silencing in reducing cell survival induced by ROS [9]. This section focuses on how IFI6 inhibits mitochondria, as an apoptotic signaling hub, from transmitting death instructions to cells, whereas the physiological function regulation of IFI6 on mitochondria as energy metabolic organelles will be detailed in the mitochondrial function regulation section.

Inhibition of caspase activation. In TRAIL-induced apoptosis models, IFI6 overexpression significantly reduced the activation and cleavage of caspase-8 and caspase-3 under the regulation of downstream cell effects and signal pathways. In dengue virus-infected vascular endothelial cells, IFI6 overexpression inhibited caspase-3 and caspase-9 activity. Notably, the inhibitory effect of IFI6 on caspases is relatively limited, while its inhibitory effect on caspase-9 is more significant, revealing that IFI6 mainly targets the mitochondria-mediated intrinsic apoptotic pathway rather than the death receptor-mediated extrinsic apoptotic pathway [7].

Activation of JAK/STAT and PI3K-AKT signaling pathways. IFI6 can activate the JAK/STAT signaling pathway, promoting STAT1 and STAT3 phosphorylation. Phosphorylated STAT3 translocates to the nucleus and upregulates the transcription of anti-apoptotic genes (such as Bcl-2, Bcl-xL, etc.). In gastric cancer cells, IFI6 upregulates Bcl-2 expression through the JAK/STAT3 pathway, inhibiting mitochondria-mediated apoptosis. Studies have shown that both IFI6 knockdown and overexpression alter estrogen synthesis, cell proliferation, and apoptosis in granulosa cells. IFI6 can activate the PI3K-AKT signaling pathway, upregulate activator protein 1, promote estrogen synthesis and cell proliferation, and simultaneously inhibit apoptosis [10]. In addition, IFI6 is positively correlated with the nuclear factor kappa B (NF-κB) pathway, and NF-κB activation slows the cell cycle, induces anti-apoptotic proteins and cell stemness, and enhances tumorigenicity and chemotherapy resistance [6].

Regulation of ER stress. The localization of IFI6 on the ER membrane suggests that it may participate in the regulation of ER stress. In esophageal squamous cell carcinoma, IFI6 knockdown increases the expression of ER stress markers, including glucose-regulated protein 78, C/EBP homologous protein, and X-box binding protein 1 (XBP-1), spliced form XBP-1s, accompanied by a significant increase in intracellular calcium levels. ER calcium release to mitochondria leads to mitochondrial calcium overload, which in turn induces opening of the mitochondrial permeability transition pore and cell apoptosis. This mechanism reveals the important role of IFI6 in ER–mitochondrial calcium signaling crosstalk and explains why IFI6 knockdown simultaneously activates both ER stress and mitochondrial apoptosis pro-apoptotic pathways [9].

High IFI6 expression upregulates gene sets related to cell proliferation and negatively regulates apoptosis [9]. Studies have shown that IFI6 knockdown reduces cell survival and colony formation while inducing apoptosis through upregulation of the p53/p21 signaling pathway [11]. IFI6 can regulate the radiation sensitivity of human skin cells through complex mechanisms. IFI6 is highly expressed in irradiated human and monkey skin tissues and can provide radiation protection for skin cells against radiation-induced apoptosis and oxidative stress damage. This process appears to be mediated by IFI6 participating in the regulation of heat shock transcription factor 1-mediated processes to enhance skin cell radiation protection. Similarly, IFI6 confers radioresistance in cancer cells [12]. IFI6 downregulation leads to spontaneous apoptosis of keratinocytes, revealing the anti-apoptotic properties of IFI6 in non-malignant cells [13].

#### 3.1.2. Inhibition of Ferroptosis

Ferroptosis is an iron-dependent lipid-peroxidation-driven form of programmed cell death. IFI6 overexpression leads to elevated levels of ferroptosis-inhibitory proteins solute carrier family 7 member 11 (SLC7A11) and glutathione peroxidase 4 (GPX4), thereby enhancing cellular antioxidant capacity, reducing lipid peroxidation levels, and inhibiting ferroptosis [14,15].

### 3.2. Regulation of the Immune System

IFI6 can regulate the activation of RIG-I. RIG-I is an important sensor for viral infection and can recognize 5’-triphosphorylated single-stranded RNA and short double-stranded RNA. After ligand recognition, RIG-I interacts with mitochondrial antiviral signaling protein (MAVS) on mitochondria through its N-terminal caspase recruitment domain, initiating downstream signaling cascades and inducing the production of IFN and inflammatory proteins. In addition, the RIG-I-like receptor signaling pathway, which detects viral infections and activates immune responses, is highly correlated with IFI6 [16]. IFI6 overexpression reduces the ubiquitination level of RIG-I, thereby inhibiting RIG-I activation and downstream IFN-β production. Conversely, IFI6 knockdown or knockout enhances RIG-I ubiquitination, IFN-β transcription, and ISG expression. Therefore, IFI6 acts as a negative feedback regulator of the RIG-I signaling pathway [17].

IFI6 inhibition may reduce immunosuppressive signals and reshape macrophage or T cell populations by affecting mitochondrial reactive enzymes and IFN signaling pathways [18]. Studies have shown that IFI6 expression is positively correlated with regulatory T cells (Tregs), a population critical for maintaining immune tolerance and dampening anti-tumor immunity. The abundant infiltration of Tregs in tumor tissues is frequently associated with poor prognosis in cancer patients, which may partly explain why elevated IFI6 expression correlates with unfavorable clinical outcomes. IFI6 has a significant correlation with CD4^+^ T cells and B cell receptors (BCR), and CD4+ T cells perform important functions in coordinating the immune system. In addition, IFI6 also shows significant correlation with CD8A and CD8B cells, which act as co-receptors for T cell receptors (TCR). Moreover, IFI6 expression is negatively correlated with naive B cells and plasma cells, implying that high IFI6 levels may interfere with the recruitment and activity of B cell subsets in the tumor microenvironment, thereby facilitating tumor progression and contributing to an immunosuppressive microenvironment [18]. Therefore, high IFI6 expression may affect the function of TCR and BCR, thereby affecting the body’s immune response and tumor cell infiltration and immune surveillance, but the specific mechanism remains unclear [16,18].

### 3.3. Regulation of Mitochondrial Function

As detailed above, IFI6 exerts anti-apoptotic effects by inhibiting the mitochondrial apoptotic pathway. This section discusses the physiological function regulation of IFI6 on mitochondria as the cellular energy metabolism center. Studies have demonstrated that IFI6 downregulation leads to mitochondrial Ca^2+^ uniporter-mediated mitochondrial calcium overload, inhibiting mitochondrial supercomplex assembly and respiratory phosphorylation efficiency, and increasing mtROS generation [19]. IFI6 can both inhibit and promote reactive oxygen species (ROS) production, with specific effects depending on cell type and experimental conditions. In addition, IFI6 regulates the assembly and function of mitochondrial respiratory chain complexes. In IFI6-knockdown cells, the activities of mitochondrial respiratory chain complexes I, III, and IV are reduced, the mitochondrial oxygen consumption rate is decreased, and cells rely more on glycolysis for ATP production [20].

## 4. The Multifaceted Roles of IFI6 in Diseases and Infections

In the previous section, the physiological functions of IFI6 were described. At the same time, abnormal expression of IFI6 is associated with various diseases such as neurodegeneration, autoimmunity, tumors, and viral infections, and is related to disease activity, severity, drug resistance, and outcomes. The following section will discuss the possible roles or confirmed clinical or relevant evidence that IFI6 may play or has been proven in various types of diseases and viral infections (Table 1).

### 4.1. Neurological Diseases

Aicardi–Goutières syndrome (AGS) is a group of rare disorders that predominantly affect the nervous system and skin, caused by mutations in multiple genes (such as three-prime repair exonuclease 1, adenosine deaminase acting on RNA 1, IFN induced with helicase C domain 1), leading to the accumulation of abnormal DNA and RNA metabolites, which in turn activates the type I IFN pathway and causes persistent inflammatory responses. The IFN signaling gene expression score has potential as a biomarker in AGS, and a 4-gene approach was the best-performing model, which includes IFI6 [21].

Major depressive disorder (MDD) is a common psychiatric disorder characterized by high prevalence, a high recurrence rate, and limited response to existing treatments. In a study of hippocampal dentate gyrus tissue biopsy samples from MDD patients, significantly downregulated IFI6 expression was detected, accompanied by changes in the expression of other inflammation-related genes. Therefore, the downregulation of IFI6 may play an important role in MDD by regulating neuroinflammation [22].

### 4.2. Autoimmune Diseases

The hallmark of autoimmune diseases is immune system dysregulation, leading to immune responses against self-antigens, causing tissue damage and organ dysfunction. As an important downstream effector molecule of the type I IFN pathway, IFI6 shows differential expression in various autoimmune diseases and is associated with disease activity and severity.

Systemic lupus erythematosus (SLE) is a common autoimmune disease characterized by multi-organ involvement, autoantibody production, and persistent activation of the type I IFN pathway [66]. IFI6 is one of the differentially expressed genes between SLE and healthy samples, and in CD14^+^ monocytes derived from SLE patients, IFI6 expression levels correlate with lupus activity severity, suggesting that IFI6 may be a potential biomarker for detecting SLE disease activity [24,67]. Notably, a study showed that the combination of IFI6 with other genes (such as interferon induced transmembrane protein 1 (IFITM1), interferon induced protein 44 like (IFI44L), MX dynamin-like GTPase 1 (MX1), and interferon alpha inducible protein 27 (IFI27)) exhibited excellent performance in distinguishing SLE from healthy controls and other autoimmune diseases. The IFN score is an important indicator for evaluating SLE disease activity and treatment response, and IFI6 is one of the core components of this score [25,68]. Severe SLE patients can develop lupus nephritis (LN), and IFI6 is one of the 23 hub genes of LN, potentially becoming a biomarker and new therapeutic target for LN diagnosis and treatment [26].

Rheumatoid arthritis (RA) is a chronic autoimmune disease characterized by symmetric polyarticular synovitis, with arthritis and joint damage as its features. The type I IFN pathway also plays an important role in the pathogenesis of RA. IFI6 shows differential expression in RA patients, and untreated RA patients with high IFN scores at diagnosis are associated with poor clinical outcomes, suggesting that it may serve as a biomarker for RA [69].

Multiple sclerosis is an autoimmune disease mainly characterized by inflammatory demyelinating lesions in the white matter of the central nervous system. IFI6 may become a key detection marker for multiple sclerosis. It is highly expressed in platelets of multiple sclerosis patients, and in a gene expression comparison study of 8 pairs of monozygotic twins, the twin with multiple sclerosis showed significant upregulation of IFI6 compared to their healthy sibling, which may suggest that in addition to genetic factors, environmental and epigenetic factors also play important roles in the regulation of IFI6 expression [3,27].

Psoriasis is a common chronic inflammatory skin disease characterized by excessive keratinocyte proliferation and abnormal differentiation, accompanied by significant immune cell infiltration and inflammatory factor release. In psoriasis patients, IFI6 is upregulated approximately 9-fold in non-lesional epidermis and up to 400-fold in lesional epidermis, and its expression level is positively correlated with the degree of inflammation. It may participate in the development of psoriasis by inhibiting keratinocyte apoptosis. In normal epidermis, keratinocyte differentiation is accompanied by programmed cell death, which is an important mechanism for maintaining epidermal homeostasis, and IFI6 overexpression may disrupt this balance, leading to abnormal survival and excessive proliferation of keratinocytes. Mechanistically, IFI6 inhibits mitochondrial membrane depolarization and cytochrome c release, blocking the caspase activation pathway [13,28]. It is worth noting that IFI6 has the highest expression in proliferating keratinocytes, and its expression gradually decreases as cells differentiate. This is consistent with the pathological feature of excessive proliferation of keratinocytes and blocked differentiation in the epidermis of psoriasis. In psoriatic lesions, the abnormal activation of the type I IFN signaling pathway is one of the key events in the initiation of the disease, and IFI6 is an important effector molecule downstream of this pathway. A meta-analysis of the common tissue transcriptome characteristics of psoriasis and ulcerative colitis showed that IFI6, along with chemokines such as C-X-C motif chemokine ligand 9 (CXCL9), CXCL10, and CXCL11, jointly drives the abnormal activation of the IFN signaling pathway. These chemokines can recruit Th1 cells to the inflammatory site, amplifying the local inflammatory response, suggesting that IFI6 may drive the disease process in psoriasis by regulating T cell activity [13,25].

Polymyositis (PM) and dermatomyositis (DM) are common autoimmune diseases characterized by skeletal muscle damage and weakness. The type I IFN pathway plays an important role in the pathogenesis of DM and PM. In a bioinformatics analysis, IFI6 was identified as one of the five hub genes of PM/DM [29]. IFI6 expression is elevated in various immune cells of patients with melanoma differentiation-associated gene 5+ dermatomyositis (MDA5^+^ DM). Single-cell sequencing results showed that IFI6 is upregulated in CD14^+^ monocytes, CD16^+^ monocytes, dendritic cells, and multiple T cell subsets of patients with MDA5^+^ DM complicated by rapidly progressive interstitial lung disease, and is associated with disease progression [30].

Primary Sjögren’s syndrome (pSS) is a chronic autoimmune disease characterized by lymphocytic infiltration of exocrine glands such as salivary and lacrimal glands, presenting with symptoms of dry mouth and dry eyes [70]. The type I IFN pathway plays a central role in the pathogenesis of pSS. IFI6 is overexpressed in the serum of pSS patients and is one of the six specific differentially expressed genes in pSS, and it is negatively correlated with salivary flow. The high IFI6 group shows increased activation of dendritic cells, activation of CD4 memory T cells and monocytes, while the expression of IFI6 is negatively correlated with M0 macrophages and resting CD4 T cells, suggesting an active immune microenvironment. Single-cell RNA sequencing analysis indicates that IFI6 is expressed at the highest level in dendritic cells, moderately in natural killer cells and T cells, and less in B cells. These findings support the association between IFI6 expression and immune activation in pSS [31].

In systemic sclerosis (SSc), IFI6 is highly expressed in microvascular endothelial cells of patients with SSc-associated interstitial lung disease and participates in the progression of SSc vasculopathy [71]. IFI6 expression is upregulated in antiphospholipid syndrome (APS) patients and is associated with early disease onset and poor outcomes [33]. In type 1 diabetes, IFI6 expression is elevated in insulitis and may participate in immune damage to pancreatic β cells [34]. In the screening of comorbidity biomarkers for psoriasis and inflammatory bowel disease (IBD), IFI6 was also identified as one of the 23 biomarkers of IBD [72].

### 4.3. Cancers

#### 4.3.1. Tumor Microenvironment

IFI6 is mainly expressed in epithelial tumor cells and is associated with changes in immune cell composition, suggesting that it may play a role in shaping the immune microenvironment [18]. IFI6 expression is positively correlated with Tregs, and massive infiltration of Tregs in the tumor microenvironment is usually associated with poor prognosis in patients. IFI6 expression is negatively correlated with naive B cells and plasma cells. Some studies have proposed the hypothesis that high IFI6 expression may be associated with reduced recruitment and activity of B cell subsets in the tumor microenvironment, which may contribute to tumor progression [18]. In addition, IFI6 participates in the regulation of mtROS, a process that may link IFN signaling, mitochondrial metabolism, and immune regulation in the tumor microenvironment. Therefore, IFI6 may indirectly affect the expression of immunosuppressive cytokines (such as IL-10) or regulate the composition and activity of immune cells to shape the tumor microenvironment through mtROS regulation [16,18,73].

IFI6 plays a complex role in the tumor microenvironment. It can increase the anti-tumor response through immune-stimulatory factors, while on the other hand, IFI6 may inhibit immune-suppressive molecules, various pathways, and immune cells, leading to tumor progression [18]. The increase in IFI6 is associated with the induction of type 2/17 helper T cells, resulting in the production of immunosuppressive type 2 and 17-related cytokines, indicating that IFI6 has an inhibitory effect on the tumor microenvironment in esophageal squamous cell carcinoma (ESCC) [36]. Exosomes derived from tumor-associated macrophages (TAMs) can activate IFI6 transcription in TAMs through IRF9, and the exosomes secreted by TAMs deliver IFI6 to breast cancer cells, thereby promoting their proliferation, migration, and invasion. Furthermore, knockdown of IFI6 has been proven to trigger DNA replication stress and inhibit the proliferation of breast cancer cells [74]. In adult B-cell acute lymphoblastic leukemia (B-ALL), bone marrow mesenchymal stem cells act as key cells in the tumor microenvironment, the overexpression of IFI6 in mesenchymal stem cells seems to promote the proliferation of B-ALL cells, and this process may be initiated by the stromal cell-derived factor 1/C-X-C motif chemokine receptor 4 (SDF-1/CXCR4) axis of IFI6 activating the extracellular signal-regulated kinase (ERK) signaling pathway, thereby promoting the proliferation of leukemia cells [8]. In lung adenocarcinoma, a study based on single-cell spatial transcriptomics technology indicates that an epithelial cell subpopulation (E3-IFI6) marked by IFI6 and IFITM1 and other IFN response genes is significantly enriched in the high-expression group of molecular programs related to immune therapy response. The spatial distribution analysis shows that E3-IFI6 cells have significant spatial proximity to the Ma-MHC macrophage subpopulation, and they may mediate cell-to-cell interactions through the amyloid precursor protein–CD74 axis, and this signal is significantly enhanced in patients with high potential for immune therapy response. After co-culturing lung adenocarcinoma cells with IFI6-overexpressing cells with M0 macrophages, pro-inflammatory gene expression is significantly upregulated while anti-inflammatory gene expression is downregulated, indicating that IFI6-overexpressing tumor cells can drive macrophages to polarize towards a pro-inflammatory phenotype and maintain anti-tumor immune activity [39].

#### 4.3.2. Tumor Cell Proliferation, Survival, and Migration

IFI6 can promote tumor cell proliferation and survival through its classical anti-apoptotic function. In gastric cancer, IFI6 is mainly enriched in the inner mitochondrial membrane of tumor cells, inhibiting the activity of caspase-3 by inhibiting the depolarization of the mitochondrial membrane and the release of cytochrome c, thereby inhibiting the apoptosis of cancer cells. Studies have shown that IFI6 is significantly highly expressed in tumor tissues of ESCC patients and in ESCC cell lines in vitro. Knockdown of IFI6 can inhibit the proliferation of ESCC and induce apoptosis by increasing ROS accumulation. Moreover, high expression of IFI6 can inhibit the apoptosis of tumor cells by suppressing the characteristics of IFN-1, activate the redox signal induced by immune endocrine, and then weaken the toxicity of the apoptosis pathway induced by high mtROS, thereby interfering with mtROS-mediated apoptosis [9]. In ESCC, inhibition of IFI6 can enhance the uptake of calcium by mitochondria, accumulate ROS, and induce apoptosis [75]. In tongue squamous cell carcinoma (TSCC), when IFI6 is ectopically expressed, it stabilizes the mitochondrial membrane potential, which can inhibit caspase-mediated apoptosis. The high expression of IFI6 can counteract the inhibitory effect of overexpression of activating transcription factor 3 (ATF3) on tumor cell proliferation and migration. At the same time, the expression of IFI6 can counteract the changes in AKT or ERK activity caused by the absence or overexpression of ATF3 [40]. In breast cancer cells, silencing the expression of IFI6 can trigger apoptosis in BT-549 cells and inhibit the growth of the ER-positive breast cancer cell line MCF-7 [18]. Moreover, studies have shown that IFI6 is a key downstream effector molecule of ubiquitin conjugating enzyme E2 T (UBE2T), and UBE2T inhibition leads to the downregulation of IFI6 expression, which in turn induces DNA replication stress, cell cycle arrest, and apoptosis. In breast cancer cells with UBE2T knockdown, supplementing IFI6 can partially restore cell growth [76]. In triple-negative breast cancer, the mitochondrial aspartate/glutamate carrier, solute carrier family 25 member 13, enhances complex I-dependent oxidative phosphorylation and limits mtROS, promoting STAT3 activation and nuclear translocation, and nuclear STAT3 directly induces IFI6 transcription, while IFI6 maintains mitochondrial function, limits lipid peroxidation and Fe^2+^ accumulation to confer ferroptosis resistance to tumor cells [77]. Candidate genes with epigenetic upregulation, IFI6, have been confirmed to be upregulated in cutaneous squamous cell carcinoma (cSCC) and are believed to play an important role in promoting cell proliferation, tumor cell invasion and migration [41]. In pancreatic cancer, upregulation of IFI6 can inhibit its apoptosis and promote cell proliferation [16,43]. In human multiple myeloma cell lines, IFI6 can temporarily antagonize TRAIL-induced apoptosis by maintaining mitochondrial integrity, prevent the release of pro-apoptotic factors (such as cytochrome c, Smac/DIABLO, etc.), and inhibit TRAIL-induced caspase cleavage through the death signal complex. IFI6 can also regulate the balance of Bcl-2/Bim expression to stabilize mitochondria and resist apoptosis. Knockdown of IFI6 reduces the survival rate of colorectal cancer cells and induces apoptosis by upregulating p53, p21 and BCL2-associated X [11]. IFI6 is upregulated in melanoma and its subgroup neuroblastoma rat sarcoma (NRAS)-mutated melanoma, and its expression is related to the NRAS mutation status. Knockdown of IFI6 can lead to DNA replication stress through E2F transcription factor 2 regulation of DNA replication, thereby inhibiting melanoma growth through senescence or induction of apoptosis [5].

IFI6 can promote tumor cell migration and invasion by regulating mtROS and actin cytoskeleton and reformation. In breast cancer, IFI6 can regulate the production of mtROS, triggering the action of multiple pathways, which further promote the rearrangement of the F-actin cytoskeleton and the formation of migration structures, enhancing the metastasis and invasion of cells [44,73]. Studies using lentiviral barcodes combined with single-cell RNA sequencing technology further revealed that metastatic breast cancer originates from a rare metastasis-promoting clone in the primary tumor, characterized by extracellular matrix remodeling and the activation of the dsRNA-IFN1 signaling pathway, and silencing IFI6 can significantly inhibit in vitro migration and in vivo metastasis [78]. In renal cell carcinoma (RCC), STX17 divergent transcript (STX17-DT) promotes tumor cell migration and invasion by upregulating the expression of IFI6 [15].

#### 4.3.3. Drug Resistance and Prognosis

IFI6 is closely related to resistance to various chemotherapeutic drugs and targeted drugs, and is an important molecule in tumor resistance research. At the same time, high expression of IFI6 is significantly associated with poor prognosis in various tumors. IFI6 shows high expression in gastric cancer and esophageal cancer, and is associated with tumor invasiveness and poor prognosis. In esophageal cancer, a high expression level of IFI6 is related to a lower tumor stage and an increased risk of lymph node metastasis, and it may be associated with the TP53 mutation status [16]. IFI6 overexpresses under the influence of the activator of heat shock 90 kDa protein ATPase activity 1 (AHSA1)–heat shock protein 90 alpha family class A member 1(HSP90AA1) complex, enhancing the drug resistance of epidermal growth factor receptor (EGFR)-mutated lung adenocarcinoma cells. This is related to IFI6 stabilizing mitochondrial function, reducing cell apoptosis and promoting the survival of lung adenocarcinoma cells. AHSA1 and HSP90AA1 prolong the half-life of IFI6 by reducing its ubiquitination-mediated degradation, and IFI6 promotes cell survival by inhibiting the activation of caspase-3/7 and maintaining mitochondrial membrane potential [38]. At the same time, IFI6 is associated with the drug resistance of various tumors, such as with oxaliplatin resistance in colorectal cancer, docetaxel resistance in prostate cancer, and tamoxifen resistance in breast cancer. The specific mechanisms of drug resistance need further study [14]. High expression of IFI6 is associated with poor prognosis in breast cancer patients with ER-positive, PR-positive, HER2-positive and lymph node-positive subtypes of breast cancer [18]. In hematological tumor research, the abnormally expressed IFI6 in multiple myeloma is an important factor leading to the chemical resistance of myeloma cells [8]. In colorectal cancer, the downregulation of IFI6 has been reported to activate the ROS-induced p38 mitogen-activated protein kinase (MAPK) pathway, affecting its resistance to oxaliplatin [44]. In glioma, IFI6 enhances the resistance of tumor cells to temozolomide by inhibiting ferroptosis. The overexpression of IFI6 leads to an increase in the levels of ferroptosis inhibitor proteins SLC7A11 and GPX4, reducing lipid peroxidation levels and decreasing cell death, while BCL11 transcription factor A, as the transcriptional inhibitor of IFI6, can promote ferroptosis by downregulating IFI6 and restoring the sensitivity to temozolomide [14]. In glioblastoma, microglia promote the resistance of tumor cells to temozolomide by inducing the expression of IFI6 and other ISGs [46]. Studies have shown that IFI6 is positively correlated with the NF-κB pathway. In ovarian cancer, IFI6 may affect cancer cell proliferation, invasiveness, and drug resistance through the NF-κB pathway [6]. In RCC, STX17-DT regulates the stability of IFI6 mRNA by recruiting and binding hnRNPA1, thereby reducing mtROS accumulation and attenuating ferroptosis, while IFI6 overexpression inhibits STX17-DT knockdown-induced ferroptosis, enhancing RCC resistance to axitinib [15]. IFI6 affects the radiosensitivity of tumors by regulating mtROS levels. In irradiated skin cells, the increase in IFI6 levels provides radiation protection for the cells, but the mechanism needs further clarification [12].

### 4.4. Viral Infection

Flaviviruses, such as yellow fever virus (YFV), West Nile virus (WNV), dengue virus (DENV), and Zika virus (ZIKV), induce the formation of characteristic single-membrane invagination structures, namely replication organelles, on the ER membrane after infecting host cells. These structures provide physical scaffolds and shielding environments for viral RNA replication, protecting viral RNA from degradation by host nucleases and recognition by innate immune sensors [4,51]. IFI6 is located in the ER and has previously been shown to inhibit flavivirus amplification and infection by impeding the formation of replication organelles [57]. In IFI6-overexpressing cells, viral RNA replication levels and viral protein expression are significantly reduced after infection, while viral entry and primary translation are unaffected. Meanwhile, studies have found that virus-induced ER membrane invagination is significantly reduced in IFI6-overexpressing cells, suggesting that IFI6 plays a role in the formation and initiation stages of viral replication organelles, supporting the claim that IFI6 has an antiviral effect during the early ER stage of the virus life cycle. This mechanism gives IFI6 broad-spectrum anti-flavivirus activity and makes it difficult to produce viral escape mutations. Due to the unique viral specificity of IFI6, its mechanism is likely specifically targeted at the ER single-membrane invagination induced by flaviviruses, but it cannot act on the ER-derived double-membrane vesicles formed during the replication of hepatitis C virus (HCV), which also belongs to the Flaviviridae, and coronaviruses [4]. The interaction of IFI6 with binding immunoglobulin protein (BiP) also plays an important role in its antiviral function. After BiP knockout, IFI6 protein levels significantly decrease, and IFI6 antiviral activity is lost. ATPase-deficient BiP mutants cannot restore IFI6 expression and antiviral function [4]. In addition, IFI6 overexpression can also inhibit DENV-induced vascular endothelial cell (VECs) apoptosis and ZIKV infection-related cell death through caspase-dependent pathways. These results may suggest a possibility: when expressed before infection, IFI6 can prevent infection, and when expressed after infection, IFI6 may reduce the cell death induced by the infection [49,50,51].

IFI6 has an inhibitory effect on hepatitis B virus (HBV). IFI6 can delay cytochrome c release, block HBV-specific CD8^+^ T cells from transmitting apoptotic signals through Bim, and after detaching from antigen-induced apoptosis, CD8^+^ T cells proliferate and differentiate into activated CD8^+^ T cells to clear HBV infection [79]. Moreover, in vitro and in vivo experiments have shown that IFI6 can inhibit HBV replication by specifically binding to the EnhII/Cp region [52]. There is some controversy regarding the effect of IFI6 on HCV. IFI6 can inhibit the entry and replication of HCV by inhibiting the function of EGFR kinase and the interaction between CD81 and Cludin1 (CLDN1), thereby exerting inhibitory effects on HCV [54]. However, a study reported that overexpression of IFI6 actually promoted HCV replication in cell culture and weakened the antiviral activity of IFN-α against HCV. Additionally, another study found that the p7 ion channel protein of HCV can co-precipitate with IFI6, and the overexpression of p7, as a key immune escape protein, can inhibit the antiviral function of IFI6 by depolarizing mitochondrial potential [80,81].

In chicken embryo fibroblasts, overexpression of IFI6 significantly inhibits the replication of avian reovirus (ARV), and overexpression upregulates the expression of MDA5, MAVS, and stimulator of interferon genes (STING), and downregulates the expression of interferon regulatory factor 1 (IRF1) and IFN-β, suggesting that IFI6 may affect the replication of avian reovirus by regulating the innate immune signaling pathway [2]. IFI6 inhibits Ebola virus (EBOV), and it has been identified as one of the ISGs that can significantly reduce the titer of EBOV. IFI6 inhibits the infection of EBOV from the aspects of virus entry, replication, transcription, and virus particle formation, which may be due to the direct or indirect interaction of IFI6 with the viral nuclear protein NP, but the specific mechanism needs to be further clarified [55]. In peripheral blood mononuclear cells of secondary syphilis patients, the expression level of IFI6 is upregulated and can be used as a diagnostic criterion [56].

Studies have shown that after knocking down IFI6 expression, reduced production of infectious Influenza A virus (IAV) and SARS-CoV-2 was observed [17]. In a scRNAseq study, six genes (IFI44L, MX1, MX dynamin-like GTPase 2, IFN regulatory factor 7, IFI6, and ISG15) constituted the core of the anti-IAV transcriptomic tissue response within the early window of IAV infection [58]. However, reduced production of infectious IAV was observed after IFI6 knockdown, suggesting that IFI6 may also play an unexpected promoting role in the IAV life cycle. Studies have found that IFI6 is a negative regulator of innate immune responses. IFI6 reduces IFN and ISGs production by inhibiting RIG-I activation. Therefore, IFI6 knockdown relieves this negative feedback inhibition, enhancing antiviral immune responses and thereby reducing viral replication. This finding explains some seemingly contradictory results: IFI6 inhibits the replication of certain viruses when overexpressed, but also inhibits the replication of other viruses when knocked down [17]. In SARS-CoV-2 cases, IFI6 expression is associated with disease outcomes. In hospitalized patients, IFI6 expression levels are negatively correlated with disease severity; moderate cases have higher IFI6 expression than severe cases, and IFI6 shows a protective effect against SARS-CoV-2 infection [57]. In human lung tissue explant infection models, IFI6 is one of the most significantly upregulated ISGs after SARS-CoV-2 infection. However, knocking down IFI6 also reduces the production of infectious SARS-CoV-2 virions, similar to IAV. IFI6 may promote SARS-CoV-2 replication by inhibiting innate immune responses. Therefore, these findings suggest that the expression polymorphism of IFI6 should not be ignored [53]. IFI6 can participate in the antiretroviral activity of IFN-γ and inhibit the infection of vectors based on human immunodeficiency virus type 1 (HIV-1), but in another study, it was shown that IFI6 has no effect on HIV-1 infection in Jurkat T cells, suggesting that further research is needed to prove whether the antiviral activity of IFI6 depends on the selected cell line [59]. Knockdown of IFI6 inhibits the replication of human bocavirus type 1 (HBoV1), which may be related to the apoptosis of infected cells caused by the silencing of IFI6 [60].

### 4.5. Other Diseases

Differential expression of IFI6 is associated with obese asthma. In CD4^+^ T cells of obese asthma patients with low type 2 inflammation, the expression of IFI6 and other IFN signaling pathway-related genes is significantly different from normal controls. IFI6 may participate in the pathogenesis of obese asthma by affecting T cell metabolic reprogramming, but its specific role requires further research [61]. In the cellular heterogeneity of peripheral blood from patients with moyamoya disease, IFI6 was identified as one of the potential key genes [62]. IFI6 has also been identified as a key driver gene of Kikuchi–Fujimoto disease (KFD), a rare self-limiting histiocytic necrotizing lymphadenitis, suggesting that abnormal activation of the type I IFN pathway plays a central role in the pathogenesis of KFD [63]. Gaucher disease (GD) is a common lysosomal storage disorder. GD patients have glucocerebroside accumulation in macrophages due to glucocerebrosidase gene mutations, thereby activating immune and inflammatory responses. IFI6 upregulation may reflect activation of the type I IFN pathway, suggesting that IFI6 may serve as a monitoring indicator for GD activity [64]. IFI6 is closely related to the type I IFN signaling pathway, and type I IFN is an important mediator in the pathogenesis of atherosclerosis (AS). IFI6 may participate in the pathogenesis of AS through the type I IFN signaling pathway. In addition, IFI6 is also associated with mitochondrial dysfunction, which plays an important role in the pathogenesis of AS [32]. Hypertensive nephropathy (HTN) is associated with high IFI6 expression. IFI6 is upregulated in glomerular endothelial cells and may be related to glomerular hypertension and endothelial injury caused by hypertension. IFI6 may participate in endothelial cell survival and function maintenance in HTN [65].

## 5. Conclusions

Increasing evidence indicates that IFI6 is a multifunctional IFN-stimulated gene that is widely involved in various physiological processes and disease states. The dynamic distribution of IFI6 in the mitochondrial membrane and endoplasmic reticulum, its cellular localization, tissue-specific expression patterns, and interactions with other signaling molecules, discovered due to different cell types, stimulation conditions, and different localization methods in experiments, reflect the complexity of its functions. Recent studies suggest that IFI6 plays important roles in affecting cell apoptosis, immune regulation, mitochondrial metabolism, and oxidative stress. Although the mechanisms by which IFI6 participates in various diseases are not fully understood, many current studies have explored its differential expression in various neurological diseases, autoimmune diseases, tumors, and viral infections, and many studies have deeply analyzed its involvement in disease occurrence and development.

Despite these advancements, it is necessary to acknowledge several limitations. Most of the mechanisms may not truly reflect the complexity of human diseases. Moreover, many conclusions rely on artificial overexpression or knockdown, potentially producing non-physiological effects that require validation under endogenous regulation. Clinically, IFI6-specific evidence remains limited, as most human data come from bioinformatic analyses or small cohorts rather than prospective, large-scale validation. Importantly, it is often unclear whether IFI6 is merely a downstream marker of IFN pathway activation or a causal mediator factor in disease progression. At present, the specific mechanisms by which IFI6 regulates viral infections and mediates disease progression remain unclear. It is particularly important to clarify its functions under different pathological conditions. Therefore, future research should employ conditional knockout mice, physiologically relevant expression systems, patient-derived organoids, and spatial multi-omics techniques to distinguish the causal contribution of IFI6 to disease progression from other broader IFN responses. Additionally, prospective clinical studies need to be conducted using standardized detection methods to determine whether IFI6 can serve as a reliable biomarker or therapeutic target in human diseases.

## Figures and Tables

**Table 1:** Clinical or relevant evidence of IFI6 associated with diseases.

Disease	Clinical or Related Evidence	Source of Evidence
Neurological diseases		
AGS	IFI6 has potential as a diagnostic biomarker in AGS [21]	Large-sample diagnostic study
MDD	IFI6 expression levels are downregulated in the hippocampus of MDD patients [22]	Patient sample expression analysis
Autoimmune diseases		
SLE	IFI6 expression levels are elevated in SLE patients and correlate with disease severity; it is a core component of the IFN score and a potential diagnostic biomarker [23,24,25]	Patient sample expression analysis and public databases
LN	IFI6 may become a biomarker and new drug target for the diagnosis and treatment of LN [26]	Bioinformatics screening
RA	IFI6 is highly expressed in RA patients [23]	Patient sample expression analysis
Multiple sclerosis	IFI6 is highly expressed in platelets of multiple sclerosis patients [3,27]	Diagnostic study
Psoriasis	IFI6 is a major IFN-inducible gene in psoriasis. IFI6 is highly expressed in psoriasis, and its expression level is positively correlated with the degree of inflammation. It may participate in the pathogenesis of psoriasis by inhibiting spontaneous keratinocyte apoptosis [13,28]	Patient sample expression analysis
PM/DM	IFI6 is one of the important hub genes of PM/DM [29]. Elevated IFI6 expression is associated with the progression of rapidly progressive interstitial lung disease in MDA5+ DM [29,30]	Multi-dataset bioinformatics integration and single-cell sequencing
pSS	IFI6 is overexpressed in pSS and negatively correlated with salivary flow, confirming IFI6 as a potential biomarker for interferon-driven immune activation in pSS [31]	Public dataset diagnostic model
SSc	IFI6 may participate in the progression of SSc vasculopathy [32]	Sample clinical tissue microarray
APS	IFI6 expression is upregulated in APS patients and is associated with early disease onset and poor outcomes [33]	Patient sample expression analysis
Type 1 diabetes	IFI6 is highly expressed and may be related to immune damage of pancreatic β cells [34]	Patient sample expression analysis
IBD	IFI6 has been identified as one of the biomarkers for IBD [35]	Patient sample expression analysis and animal model
Cancers		
ESCC	IFI6 inhibits the tumor microenvironment of ESCC [36]. IFI6 is highly expressed in both ESCC patients and cultured ESCC cells [9,15]	Cells and animal models
Breast cancer	IFI6 is associated with the tumor microenvironment in breast cancer, enhances breast cancer cell invasion and migration, and is associated with drug resistance [14,37]	Cells and animal models
B-ALL	Overexpression of IFI6 in bone marrow mesenchymal stem cells may be a key factor promoting B-ALL cell proliferation [8]	Cells and animal models
Lung adenocarcinoma	High expression of IFI6 can maintain the tumor immune activity and enhance the survival and drug resistance of lung adenocarcinoma cells [38,39]	Cells and animal models
Gastric cancer	IFI6 is mainly enriched in the inner mitochondrial membrane of gastric cancer cells, inhibiting mitochondrial membrane depolarization and cytochrome c release to inhibit caspase-3 activity, thereby inhibiting cancer cell apoptosis [7]	Gastric cancer cell line
TSCC	IFI6 expression in TSCC cells is higher than in normal tissues [40]	Cells and animal models
cSCC	IFI6 has been confirmed to be upregulated in cSCC [41]	Multimodal analysis and cell function validation
Multiple myeloma	IFI6 is abnormally expressed in multiple myeloma and is associated with chemoresistance; it antagonizes TRAIL-induced apoptosis by preserving mitochondrial integrity [42]	Multiple myeloma cell line
Pancreatic cancer	Upregulation of IFI6 expression can inhibit pancreatic cancer cell apoptosis and promote cell proliferation [16,43]	Cells and animal models
Colorectal cancer	IFI6 is highly expressed in colorectal cancer cells and is associated with poor prognosis [11,44]	Cells and clinical prognosis analysis
Melanoma	IFI6 is overexpressed in patient melanoma samples [45]	Patient sample expression analysis
NRAS mutant melanoma	Knockdown of IFI6 inhibits cell transformation and melanoma growth [5]	Cells and animal models
Glioma	IFI6 overexpression inhibits ferroptosis in glioma cells, increasing drug resistance and survival rate [14]	Cells and animal models
Glioblastoma	IFI6 shows highly significant and differential expression in glioblastoma cells [46]	Cells
Prostate cancer	IFI6 is associated with immune infiltration and drug resistance in prostate cancer [14,47]	Bioinformatics and public databases
Ovarian cancer	IFI6 is positively correlated with the NF-κB pathway, affecting ovarian cancer cell proliferation, invasiveness, and drug resistance [6]	Single-cell RNA-seq and cells
RCC	IFI6 overexpression restores axitinib resistance in long non-coding RNA STX17-DT-knockdown RCC cells [15]	Cells and animal models
DT	Upregulation of IFI6 may contribute to DT cell survival and invasion and is associated with the prognosis of stratified DT [48]	Clinical pathological analysis
Viral infections		
YFV	High IFI6 expression inhibits YFV-induced ER invagination, thereby inhibiting YFV [49]	Cells
WNV	IFI6 has an inhibitory effect on WNV [4]	Cells
DENV	IFI6 inhibits DENV-induced VEC apoptosis through a caspase-dependent pathway [49,50]	Cells
ZIKV	IFI6 overexpression may inhibit ZIKV infection-related cell death [51]	Cells
HBV	High IFI6 expression can inhibit HBV replication and gene expression [2,52]	Cells and animal models
HCV	IFI6 can inhibit HCV entry and replication [53,54]	Cells
ARV	IFI6 overexpression inhibits ARV replication [2]	Cells
EBOV	IFI6 has an inhibitory effect on EBOV transcription and replication [55]	ISG screening and cells
Secondary syphilis	IFI6 is upregulated in peripheral blood mononuclear cells of patients with secondary syphilis and can have diagnostic value [56]	Patient sample expression analysis
SARS-CoV-2	In hospitalized patients, IFI6 expression levels are higher in moderate cases than in severe cases, and IFI6 expression is associated with lower mortality risk and IMV [53,57]	Patient sample expression analysis
IAV	IFI6 has been identified as one of the core transcriptomic response mRNAs to IAV infection [58]	Animal models
HIV-1	IFI6 can inhibit HIV-1-based vector infection [59]	Cells
HBoV1	IFI6 inhibition significantly reduces HBoV1 replication [60]	Cells
Other diseases		
Obese asthma	Differential expression of IFI6 is associated with obese asthma [61]	Comorbidity bioinformatics
Moyamoya disease	IFI6 is one of the potential key genes for the heterogeneity of peripheral blood cells in Moyamoya disease [62]	Comorbidity bioinformatics
KFD	IFI6 has been identified as a key seed gene for KFD [63]	Comorbidity bioinformatics
GD	IFI6 is significantly highly expressed in GD [64]	Comorbidity bioinformatics
AS	IFI6 may be involved in the pathogenesis of AS through the type I IFN signaling pathway [32]	Comorbidity bioinformatics
HTN	IFI6 is upregulated in the endothelial cells of HTN [65]	Cells

AGS: Aicardi–Goutières syndrome, MDD: major depressive disorder, SLE: systemic lupus erythematosus, IFN: interferon, LN: lupus nephritis, RA: rheumatoid arthritis, PM/DM: polymyositis/dermatomyositis, MDA5: melanoma differentiation-associated gene 5, pSS: primary Sjogren’s syndrome, SSc: systemic sclerosis, APS: antiphospholipid syndrome, IBD: inflammatory bowel disease, ESCC: esophageal squamous cell carcinoma, B-ALL: adult B-cell acute lymphoblastic leukemia, TSCC: tongue squamous cell carcinoma, cSCC: cutaneous squamous cell carcinoma, NRAS: neuroblastoma rat sarcoma, NF-κB: nuclear factor kappa B, RCC: renal cell carcinoma, STX17-DT: STX17 divergent transcript, DT: desmoid tumor, YFV: yellow fever virus, ER: endoplasmic reticulum, WNV: West Nile virus, DENV: dengue virus, VECs: vascular endothelial cell, ZIKV: Zika virus, HBV: hepatitis B virus, HCV: hepatitis C virus, ARV: avian reovirus, EBOV: Zaire Ebola virus, IMV: invasive mechanical ventilation, IAV: influenza A virus, HIV: human immunodeficiency virus, HBoV1: human bocavirus 1, KFD: Kikuchi–Fujimoto disease, GD: Gaucher disease, AS: atherosclerosis, HTN: hypertensive nephropathy.

## Data Availability

No new data were created or analyzed in this study. Data sharing is not applicable to this article.
